# Association between Relative Thrombocytosis and Microalbuminuria in Adults with Mild Fasting Hyperglycemia

**DOI:** 10.3390/jpm14010089

**Published:** 2024-01-12

**Authors:** Jong Wook Choi, Tae Hoon Kim, Joon-Sung Park, Chang Hwa Lee

**Affiliations:** 1 Research Institute of Medical Science, Konkuk University School of Medicine, Chungju 27478, Republic of Korea; sodasoo@empas.com; 2 Department of Internal Medicine, CHA Bundang Medical Center, Seongnam 13495, Republic of Korea; happyto21@gmail.com; 3 Department of Internal Medicine, Hanyang University College of Medicine, Seoul 04763, Republic of Korea; changhwa@hanyang.ac.kr

**Keywords:** prediabetic state, platelet count, hemoglobin A1c, albuminuria

## Abstract

An elevated platelet count may contribute to significant thrombotic events and pose a risk for diabetic microvascular complications. Albuminuria, one of the hallmarks of diabetes, is thought to be a risk factor for endothelial dysfunction. In this study, we investigated the association between relative thrombocytosis and an increased urine albumin-to-creatinine ratio in healthy adult participants. Using multivariate analyses on data from the Korea National Health and Nutrition Examination Survey V–VI, 12,525 eligible native Koreans aged ≥ 20 were categorized into platelet count quintiles by sex. The highest platelet count quintile included younger, more obese participants with elevated white blood cell counts, poor lipid profiles, and a better estimated glomerular filtration rate. Restricted cubic spline regression analysis revealed significant associations between platelet count and fasting blood glucose, glycated hemoglobin, and urine albumin-to-creatinine ratio. Adjusted logistic regression models indicated that heightened fasting blood glucose and platelet count were linked to risk of microalbuminuria (fasting blood glucose, odds ratio = 1.026, 95%CI = 1.011–1.042; platelet count, odds ratio = 1.004, 95%CI = 1.002–1.006). Particularly, an increased platelet count was notably associated with microalbuminuria progression in subjects with impaired fasting glucose. These findings suggest that an elevated platelet count, even below diagnostic thrombocytosis levels, independently correlates with an increased risk of vascular endothelial dysfunction in patients with impaired fasting glucose.

## 1. Introduction

Diabetes mellitus (DM) can be accompanied by various systemic complications and is often caused by metabolic abnormalities in vascular functions and responses [1]. The risk of the development and progression of DM-related vascular complications increases with the duration and severity of hyperglycemia [2]. Several experimental studies on the pathogenesis of diabetic vascular complications demonstrated that increased glycemic exposure contributed to the overproduction of reactive oxygen species and alteration of various intracellular signaling pathways in vascular beds, which could be responsible for the derangement of endogenous vascular protective mechanisms and the initiation of various vascular endothelial dysfunctions [3,4].

In particular, diabetic kidney disease is progressed by hemodynamic changes and inflammations occurring in the microvascular unit and is characterized by albuminuria, that is, an increase in the urinary excretion rate of albumin. Microalbuminuria, which may be prominent several years before the diagnosis of overt diabetic kidney disease, may imply the presence of glomerular filtration barrier dysfunction, a significant feature of vascular endothelial injury related to pathologic inflammatory responses in patients with DM [5,6,7]. Recent evidence suggests that microalbuminuria is a strong and independent predictor of increased cardiovascular and all-cause mortality among individuals with and without DM [8,9]. Thus, identifying the risk of albuminuria is critical for improving the long-term prognosis of DM.

Platelet activation is one of many factors related to the pathogenesis of albuminuria in DM patients and is related to endothelial dysfunction, chronic vascular inflammation, intravascular thrombi, and microangiopathy. Platelets are anucleate cytoplasmic discs derived from megakaryocytes and circulate in the blood, playing critical roles in managing vascular integrity and regulating hemostasis [10,11,12]. Accumulating evidence shows that the deterioration of platelet structure and function in patients with DM could be responsible for the development of vascular complications [13,14]. Recently, some authors argued that not only long-term continuous hyperglycemic exposure but also short-term hyperglycemic spikes were deeply associated with thrombocytosis and platelet hyperactivity, which may contribute to vascular endothelial injury and blood clotting disorders [15,16,17,18,19].

To provide a mechanistic explanation of the relationship between platelets, vascular endothelial dysfunction, and diabetes, oxidative stress occurs due to insulin resistance and high blood sugar, which inhibits fibrinolysis in diabetes. Additionally, as the secretion of free fatty acids becomes excessive, endothelial dysfunction occurs, and platelet dysfunction occurs as metabolic abnormalities such as obesity and dyslipidemia occur. These processes ultimately lead to the risk of vascular complications due to changes in platelet function [20]. Although increased platelet counts (PCs) as well as platelet dysfunction could be related to microvascular complications of DM, there is little clinical evidence of an association between relative thrombocytosis and vascular endothelial dysfunction, especially in the prediabetic condition. Therefore, in this study, we aimed to evaluate whether relative thrombocytosis is associated with increased albuminuria through a possible interaction with mild hyperglycemia.

## 2. Materials and Methods

### 2.1. Study Population

Data were collected from public-use data sets from the Korean National Health and Nutrition Examination Survey (KNHANES) conducted by the Korea Centers for Disease Control and Prevention (KCDC) among non-institutionalized Korean civilians between 2011 and 2014. All participants were volunteers and provided written informed consent before enrollment. All data, except survey data, were anonymized before the analysis. This study was approved by the Institutional Review Board of the KCDC (No. 2011-02CON-06-C, 2012-01EXP-01-2C, 2013-07CON-03-4C, 2013-12EXP-03-5C).

**Figure 1:**
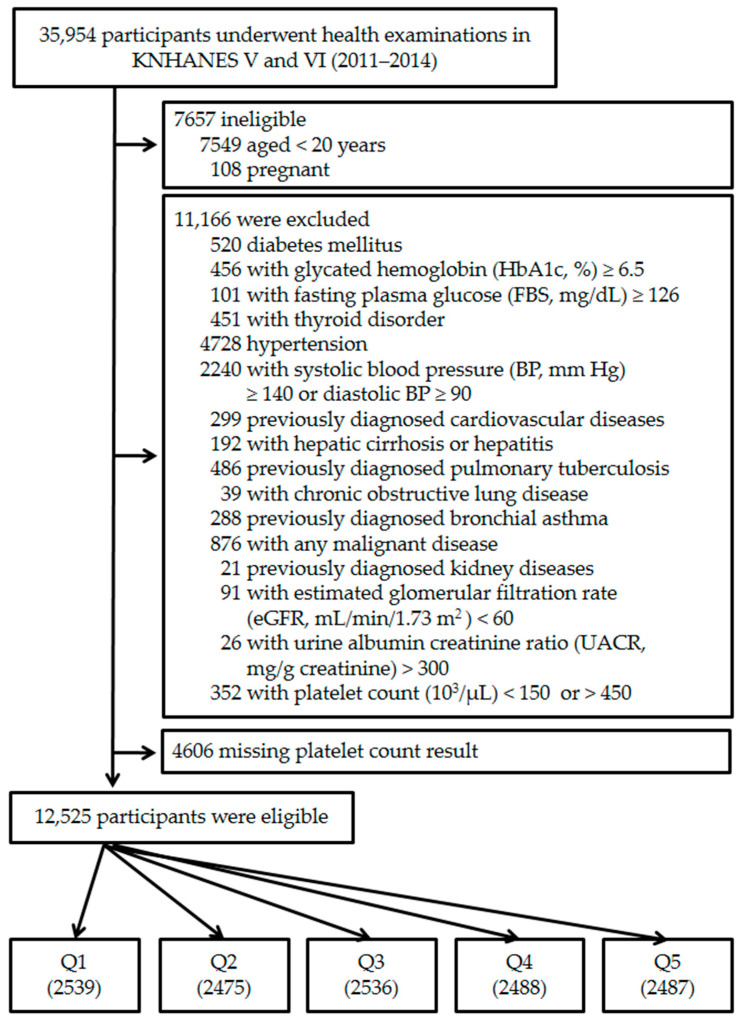
Flow chart of the study group enrollment process. KNHANES, Korean National Health and Nutritional Examination Survey; Q, platelet count quintile.

A total of 35954 individuals had participated in the KNHANES 2011–2014. The following participants were excluded from this study: those for whom data were incomplete (anthropometric or laboratory); those <20 years of age; currently pregnant women; or those who had any medical problem related to chronic cardiovascular disease, estimated glomerular filtration rate (eGFR, mL/min/1.73 m^2^) < 60, urine albumin/creatinine ratio (UACR, mg/g creatinine) > 300, or plasma PC (10^3^/µL) < 150 or >450. The final 12,525 participants were divided into five quintiles according to their PCs stratified by sex (Figure 1).

### 2.2. Anthropometric and Clinical Measurements

Anthropometric measurements were made by well-trained examiners. The participants wore lightweight gowns or underwear. Height was measured to the nearest 0.1 cm using a portable stadiometer (Seriter, Bismarck, ND, USA). Weight was measured to the nearest 0.1 kg on a calibrated balance beam scale (Giant-150N; Hana, Seoul, Republic of Korea). Waist circumference (WC) was measured using a flexible tape at the narrowest point between the lowest border of the rib cage and the uppermost lateral border of the iliac crest at the end of normal expiration. Body mass index (BMI) was calculated as weight in kilograms divided by the square of the height in meters. Smoking history was gathered through self-reported questionnaires. Smokers are individuals who smoked more than 100 cigarettes in their lifetime. Smoking duration (in years) and smoking dose (daily average) are multiplied to calculate smoking pack-years. Cut-off points of anthropometric variables are presented in the Supplemental Table S1.

Blood pressure (BP) was measured three times using a mercury sphygmomanometer (Baumanometer; Baum, Copiague, NY, USA) while subjects were in a sitting position following a 5 min rest period. The average values of the last 2 recorded systolic and diastolic BP values were used in the analysis.

### 2.3. Laboratory Tests

Venous blood samples were collected after 8 h overnight fasting. Platelets were counted using an automated hematology analyzer (Sysmex XE-2100D Hematology Analyzer, Sysmex Corporation, Kobe, Japan) in EDTA-anticoagulated whole blood samples. Fasting plasma concentrations of glucose, triglyceride (TG), high-density lipoprotein (HDL) cholesterol, and low-density lipoprotein (LDL) cholesterol were determined by a Hitachi Automatic Analyzer 7600 (Hitachi, Tokyo, Japan). Glycated hemoglobin (HbA1c) levels were determined by high-performance liquid chromatography using an automated HLC-723G7 analyzer (Tosoh Corporation, Tokyo, Japan). Serum creatinine levels were measured colorimetrically (Hitachi Automatic Analyzer 7600) and eGFR was calculated using the Chronic Kidney Disease Epidemiology Collaboration equation [21]. To obtain the UACR, urinary albumin was measured in spot urine using the immunoturbidimetric method and urinary creatinine was measured using the colorimetric method. Reference values of all laboratory tests completed are presented in the Supplemental Table S1.

### 2.4. Definitions

According to the American Diabetes Association standards of medical care from 2024 [22], mild hyperglycemia was defined as a fasting blood glucose (FBS) of 100–125 mg/dL or an HbA1c of 5.7–6.4% without the use of hypoglycemic medications. According to the KDIGO 2012 Clinical Practice Guideline [23], participants with microalbuminuria were defined as those with a UACR of 30–300 mg/g creatinine.

### 2.5. Statistical Analysis

All data, including sociodemographic data, medical conditions, anthropometric and clinical measurements, and laboratory results, are presented as mean ± SE or frequencies (proportions). Data were analyzed using sampling weights to account for multistage and stratified sampling. The normality of the distribution was ascertained by the Kolmogorov–Smirnov test. If variables did not follow a normal distribution, logarithmic (log) transformation was applied before the statistical analysis. Participant characteristics were analyzed based on platelet count quintiles using weighted one-way analysis of variance (ANOVA) tests for continuous variables and weighted chi-square tests. The generalized linear model was used to compare quantitative variables and the chi-squared and Fisher’s exact test to compare proportions for the categorical variables. Odds ratios (ORs) with 95% confidence intervals (CIs) were calculated in multiple logistic regression models according to the presence of a dependent variable (case vs. control) after controlling for potential confounding factors within platelet count quintiles. Restricted cubic spline (RCS) regression analysis was used to find the possible non-linear dependency of the association between candidate risk factors and increased risk of dependent variable [24]. A two-tailed *p* < 0.05 was considered statistically significant. Finally, we repeated the statistical analysis and analyzed the significant results obtained. Statistical Analysis Software version 9.4 (SAS Institute Inc., Cary, NC, USA) was used for all of the analyses.

## 3. Results

### 3.1. Baseline Characteristics

The participants (*n* = 12,525) comprised 4939 men and 7586 women with a mean age of 44.0 ± 14.5 years. They were divided into quintiles according to PCs stratified by sex. Participants in the highest PC quintile were likely to be younger and more obese and have increased white blood cell counts, greater glycemic exposure, a poor lipid profile, and better eGFR compared to those in the lower quintiles. However, there was a marginal difference in UACR among PC quintiles (Table 1).

### 3.2. Relation of Increased PC with Microalbuminuria and Related Risk Factors

In conventional linear regression analysis, we found that although PC was significantly related to BMI, white blood cell count, hemoglobin, eGFR, FBS, HbA1c, TG, LDL cholesterol, 25-vitamin D, and UACR, there was no significant relation of PC with WC, systolic BP, diastolic BP, and HDL cholesterol (Table 2).

**Figure 2:**
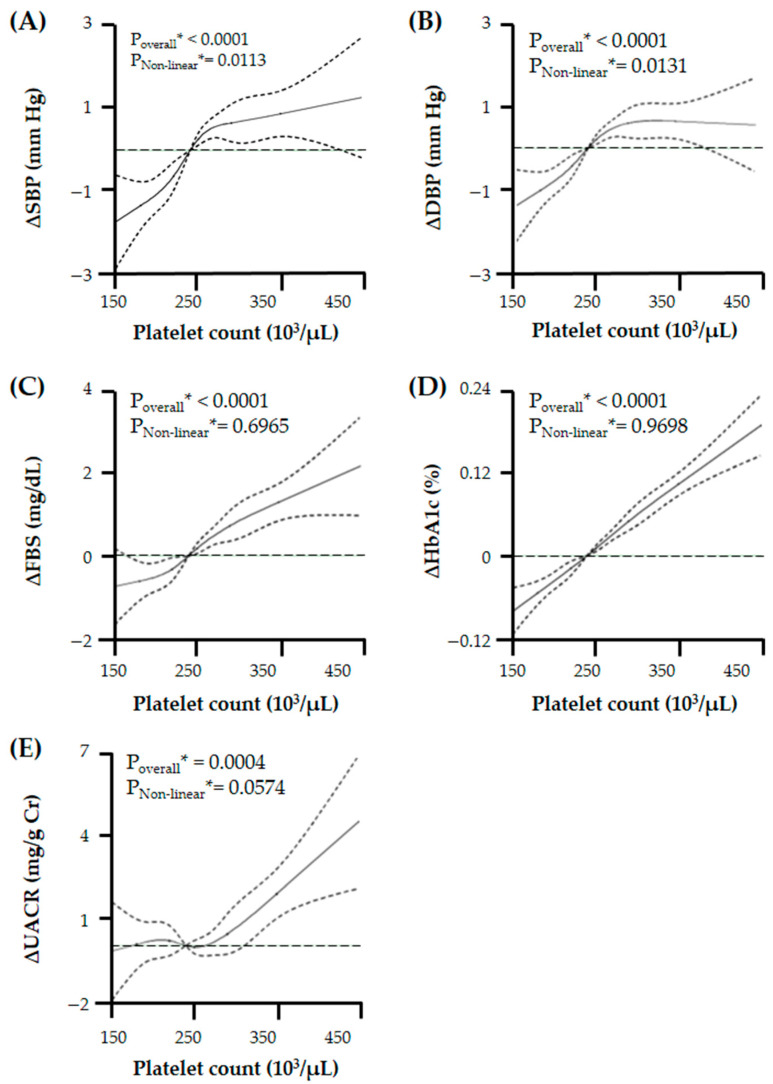
Relationship of platelet count with changes in (**A**) systolic blood pressure (BP), (**B**) diastolic BP, (**C**) fasting blood glucose (FBS), (**D**) glycated hemoglobin (HbA1c), and (**E**) urine albumin/creatinine ratio (UACR) with a chosen reference platelet count of 240 × 10^3^/μL. There is a varied relationship between platelet count and UACR or related clinical risk factors. Especially, platelet count has a J-shaped relation with UACR. Solid lines represent changes in microalbuminuria and related clinical risk factors and dashed lines represent 95% confidential intervals. * Calculated by restricted cubic spline (RCS) regression adjusted for age, sex, and smoking history.

To evaluate the relationship pattern of PC with candidate risk factors of vascular endothelial dysfunction, we performed a RCS linear regression model with age, sex, and smoking history as covariates and found that PC had differing relationships with these risk factors: non-linear relationship with BPs, linear relationship with indicators of glycemic exposure, and potential J-shaped relationship with UACR (Figure 2).

### 3.3. Association of Relative Thrombocytosis with Microalbuminuria

We performed multiple logistic regression models, using age, sex, and smoking history as covariates, to find a possible association between PC and other candidate predictors for vascular endothelial dysfunction. When participants with microalbuminuria were compared to controls, we found that both PC and FBS were significantly associated with microalbuminuria, and further adjustment for systolic BP, FBS, aspartate aminotransferase, triglyceride, and LDL cholesterol as predictors did not attenuate these associations (PC, adjusted OR = 1.002, 95% CI = 1.001–1.004; FBS, adjusted OR = 1.026, 95% CI = 1.011–1.042; Table 3).

**Figure 3:**
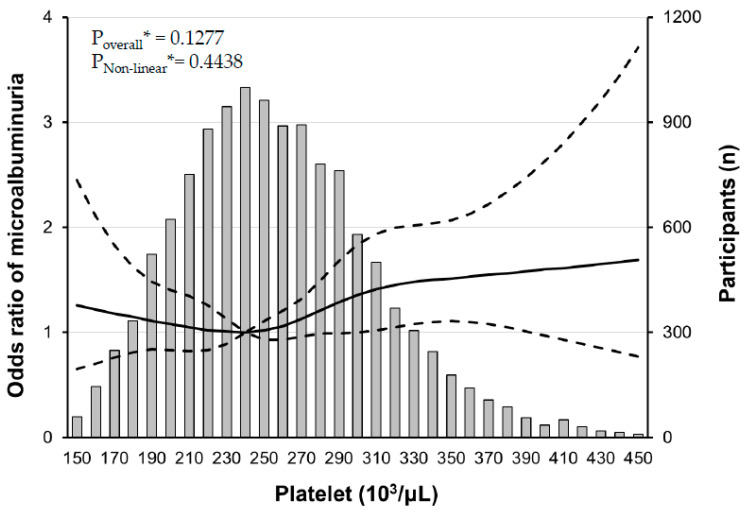
Multiple logistic regression analysis of microalbuminuria^#^-specific risk with restricted cubic splines (RCS). There is a non-linear association between platelet count and the risk of microalbuminuria. The solid line represents the risk of albuminuria and dashed lines represent 95% confidential intervals. RCS plots are performed using a platelet count of 240 × 10^3^/μL as a reference. * Calculated by RCS logistic regression model adjusted for age, sex, smoking history, systolic BP, AST, triglyceride, and LDL cholesterol. ^#^ defined as a urine albumin/creatinine ratio between 30 and 300 mg/g creatinine.

**Figure 4:**
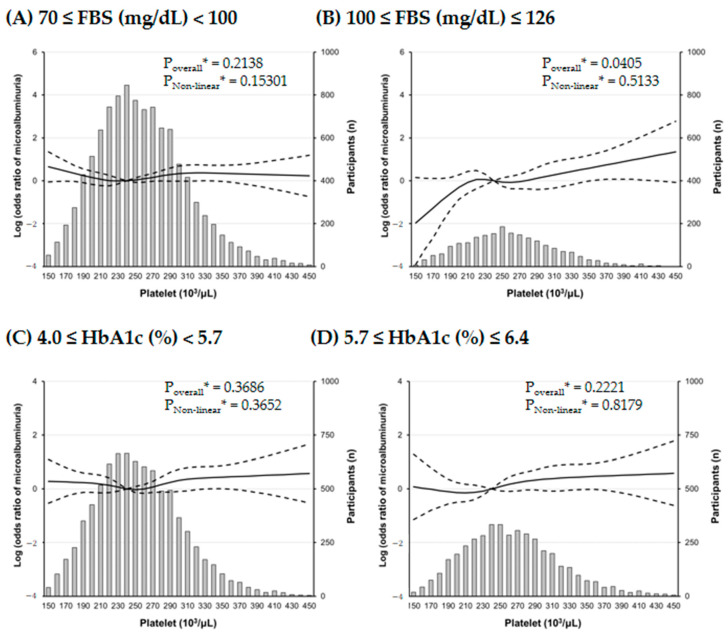
Multiple logistic regression analysis of microalbuminuria^#^-specific risk with restricted cubic splines (RCS) regression. The effect of platelet count on microalbuminuria prominently increases in subjects with mild fasting hyperglycemia. Solid lines represent the risk of albuminuria and dashed lines represent 95% confidential intervals. RCS plots are performed using a platelet count of 240 × 10^3^/μL as a reference. * Calculated by RCS logistic regression model adjusted for age, sex, smoking history, systolic BP, AST, triglyceride, and LDL cholesterol. ^#^ defined as a urine albumin/creatinine ratio between 30 and 300 mg/g creatinine.

Our further analyses revealed that there was a J-shaped association between PC and the risk of microalbuminuria in the RCS analysis (Figure 3), and interestingly, the risk of microalbuminuria was increased with increasing PCs only in participants with mild fasting hyperglycemia, not in participants with mild elevated HbA1c (Figure 4).

## 4. Discussion

This study provides a comprehensive overview of the relationship between metabolic disturbance, relative thrombocytosis, and microvascular endothelial dysfunction, showing that an elevated PC in participants with mild hyperglycemia is associated with an increased risk of microalbuminuria. Such results point out that, in addition to the established risk factor of microalbuminuria, relative thrombocytosis could be an important indicator of predicting vascular endothelial dysfunction before the diagnosis of overt DM.

In the analysis based on platelet count quintiles, it became evident that there were recognizable differences in various baseline characteristics among the groups. An intriguing observation was that microalbuminuria exhibited a distinct pattern across these quintiles. Specifically, in quintile 1, microalbuminuria was higher compared to quintiles 2–4, and as platelet count quintiles increased, there was a gradual rise in microalbuminuria, reaching its highest level in quintile 5. Next, in the linear regression performed, it was confirmed that microalbuminuria had a positive correlation with platelet count. Additionally, not only glycemic index but also white blood cell count demonstrated a relatively strong positive correlation with platelet count in linear regression. Multivariate logistic regression analysis was performed to more clearly identify the risk factors for microalbuminuria. The results highlighted that systolic blood pressure, platelet count, and glycemic index were contributors to an increased risk of microalbuminuria. Interestingly, white blood cell count did not emerge as a significant risk factor for microalbuminuria in this analysis. Logistic regression model I indicated that an elevated systolic blood pressure increased the risk of albuminuria, a finding that aligns with expectations, considering microalbuminuria’s established status as a cardiovascular risk factor [25]. To explore the refined relationship between platelet count and microalbuminuria, this study employed restricted cubic splines, revealing a J curve. This non-linear relationship underscores the intricate nature of the association, implying the existence of a complex mechanism between platelet count and microalbuminuria.

Our study revealed that although a mild increase in glycemic exposure was related to an increased PC, it was more correlated with FBS than HbA1c. Long-term exposure to metabolic disturbances and the accumulation of pro-inflammatory mediators in diabetes can elicit pathologic platelet activation and cause microvascular complications [19,26,27,28,29]. Recent studies have demonstrated that an acute short-term hyperglycemic spike is also a sufficient stimulus to increase high shear stress-induced platelet activation in patients with type 2 DM [15,18]. Impaired fasting glucose due to inappropriate endogenous glucose production originates from hepatic insulin resistance, reduced hepatic glucose clearance, and dysfunction of glucose uptake and production [30]. Meanwhile, patients with an elevated HbA1c level reflecting mean glucose level during the past 2 to 3 months may have continuously worsening impaired fasting glucose, impaired glucose tolerance characterized by skeletal muscle resistance and beta cell dysfunction, or both [31]. Although little is known about whether mild fasting hyperglycemia is associated with relative thrombocytosis and related systemic complications, it can be assumed that increased PC has a greater impact on impaired fasting glucose in prediabetes rather than impaired glucose tolerance.

Our linear regression analysis showed that there was a close relationship between plasma PC and clinical parameters of chronic systemic inflammation, such as white blood cell and albuminuria, in the general population. Low-grade systemic inflammation can account for not only the activation of platelets in peripheral blood but also the proliferation and differentiation of megakaryocyte colony-forming units in bone marrow, which may affect the long-term process of atherosclerosis as well as the development of microvascular complications of various chronic illnesses [17,32]. Some authors argued that an increased PC might be one of the significant contributors to the development of microalbuminuria and overt diabetic nephropathy [16,33]. Such findings suggest that an elevated PC, even below the diagnostic criteria of thrombocytosis, could have a role in predicting the initiation of a systemic inflammatory response and related microvascular injury.

Unfortunately, we failed to find a possible relation between leukocytosis and the development of microalbuminuria in this study. This result was inconsistent with those of previous studies showing that neutrophils were glucose-sensitive inflammatory cells and played a critical role in platelet activation [18,26]. Because of the limitation of our cross-sectional study design, we did not have a differential count of white blood cells, which made it difficult to efficiently demonstrate the long-term harmful effects of neutrophil activation on the microvascular endothelium. Another possibility is that this pathologic process may result from the accumulation of more severe metabolic derangements or some confounding variables, such as other genetic factors or environmental conditions, which could have exerted a hidden effect on our research outcomes.

There were several limitations to our study. First, because this cross-sectional population-based study did not include other platelet indices, such as plateletcrit, mean platelet volume, or platelet distribution width, we had to use only PC as a surrogate marker. Second, because a very small number of participants had UACR > 300 mg/g creatinine, we could not include them in the study. This limitation made it impossible to investigate the relationship between metabolic disturbance and severely increased albuminuria. Third, our public-use data sets did not contain the results of an oral glucose tolerance test, insulin level, and associated adipokines, as well as details regarding a history of acute infection, recent surgeries, and medication usage such as corticosteroids. Consequently, we could not finely define the metabolic and inflammatory status of the participants. Fourth, there is a limitation to explaining the mechanism of an independent role of platelets in microvascular endothelial dysfunction due to a lack of data about thrombopoietin, C-reactive protein, pro-inflammatory cytokines, renin, angiotensin II, or aldosterone in this study. Furthermore, our findings could have been influenced by the anticoagulants used in the plasma samples. Fifth, because of a social desirability bias in the self-reporting of medical history, medication, and use of tobacco and alcohol, our results may conflict with those of previous studies. Finally, participants might have forgotten pertinent relevant details.

Despite the aforementioned limitations, our study was relatively successful in demonstrating a correlation between subtle changes in platelet count and the development of microalbuminuria in prediabetes. Our study results are particularly meaningful given that the literature on platelet dynamics in prediabetes is rare [34,35]. Previous studies on platelets in diabetes have revealed various pathological changes, such as changes in platelet hematocrit, platelet indices, increased intracellular calcium concentration, increased activation, adhesion, reactivity, and turnover of platelets, and increased reticular platelets [36,37,38]. These platelet abnormalities have a well-established association with the hyperglycemia, insulin resistance, and oxidative stress observed in diabetes, potentially leading to endothelial dysfunction, increased vascular inflammation, and increased risk of vascular complications [39,40,41,42]. These abnormal changes in platelets may play a major role in the development of microvascular complications of diabetes, and accumulated evidence in diabetic patients suggests that platelet changes may contribute to increased microalbuminuria [43,44,45]. Ultimately, we hope that our study will lead to a reassessment of the importance of platelet count testing in prediabetes, which has received relatively little attention.

## 5. Conclusions

In conclusion, the results of the present study showed that in the condition of impaired fasting glucose, a relative thrombocytosis was independently associated with increased risk of microalbuminuria even before the appearance of overt DM. Further study on the role of PC as a valuable predictor of diabetic vascular endothelial dysfunction is needed.

## Figures and Tables

**Table 1:** General characteristics grouped according to blood platelet count.

	Quintile 1	Quintile 2	Quintile 3	Quintile 4	Quintile 5	*p*
Platelet (10^3^/μL) in males	≥150, ≤204	>204, ≤230	>230, ≤254	> 254, ≤285	>285, ≤448	
Platelet (10^3^/μL) in females	≥150, ≤218	>218, ≤246	>246, ≤274	> 274, ≤306	>306, ≤449	
Variable	(*n* = 2539)	(*n* = 2475)	(*n* = 2536)	(*n* = 2488)	(*n* = 2487)	
Age (years)	42.9 ± 0.4	40.3 ± 0.3	40.1 ± 0.3	39.6 ± 0.3	39.1 ± 0.3	<0.0001
Sex (% male)	997 (39)	985 (40)	1015 (40)	969 (39)	976 (39)	0.3921
Smoker (%)	377 (15)	450 (18)	494 (19)	568 (23)	612 (25)	<0.0001
Body mass index (kg/m^2^)	22.7 ± 0.1	22.9 ± 0.1	23.1 ± 0.1	23.3 ± 0.1	23.7 ± 0.1	<0.0001
Waist circumference (cm)	77.6 ± 0.2	78.2 ± 0.2	78.7 ± 0.2	79.3 ± 0.3	80.0 ± 0.3	<0.0001
Systolic BP (mmHg)	110.0 ± 0.3	109.9 ± 0.3	111.0 ± 0.3	111.2 ± 0.3	111.1 ± 0.3	<0.0001
Diastolic BP (mmHg)	72.3 ± 0.2	72.7 ± 0.2	73.2 ± 0.2	73.4 ± 0.2	73.4 ± 0.2	<0.0001
White blood cell (10^9^/L)	5.36 ± 0.03	5.71 ± 0.04	6.01 ± 0.04	6.31 ± 0.04	6.78 ± 0.04	<0.0001
Hemoglobin (g/dL)	14.19 ± 0.04	14.29 ± 0.04	14.25 ± 0.04	14.24 ± 0.04	13.96 ± 0.05	<0.0001
Platelet (10^3^/μL)	191.1 ± 0.4	225.6 ± 0.2	252.0 ± 0.3	279.6 ± 0.3	332.3 ± 0.8	<0.0001
eGFR (mL/min/1.73 m)	99.3 ± 0.4	100.4 ± 0.4	101.1 ± 0.3	101.9 ± 0.4	103.1 ± 0.4	<0.0001
FBS (mg/dL)	91.2 ± 0.2	91.3 ± 0.2	91.9 ± 0.2	91.8 ± 0.2	92.4 ± 0.2	<0.0001
Hemoglobin A1c (%)	5.46 ± 0.01	5.47 ± 0.01	5.50 ± 0.01	5.51 ± 0.01	5.56 ± 0.01	<0.0001
AST (IU/L)	21.1 ± 0.4	20.7 ± 0.2	20.4 ± 0.2	20.2 ± 0.2	20.7 ± 0.3	0.1423
ALT (IU/L)	19.9 ± 0.6	19.6 ± 0.4	20.0 ± 0.2	20.0 ± 0.4	21.0 ± 0.4	0.0605
Triglyceride (mg/dL)	106.7 ± 2.2	113.8 ± 2.4	121.2 ± 2.3	126.4 ± 2.6	134.1 ± 2.6	<0.0001
HDL cholesterol (mg/dL)	52.5 ± 0.3	52.5 ± 0.3	51.7 ± 0.3	51.9 ± 0.3	51.1 ± 0.3	0.0005
LDL cholesterol (mg/dL)	107.5 ± 2.0	112.9 ± 1.8	116.7 ± 1.9	117.6 ± 1.7	120.4 ± 1.7	<0.0001
25-vitamin D (ng/mL)	17.0 ± 0.2	16.4 ± 0.2	16.7 ± 0.2	16.5 ± 0.2	16.0 ± 0.2	0.0012
UACR (mg/g creatinine)	6.5 ± 0.4	5.4 ± 0.4	5.7 ± 0.3	5.7 ± 0.3	7.0 ± 0.4	0.0573

Results are expressed as mean ± SE or frequencies (and proportions). A two-tailed *p* < 0.05 was considered statistically significant. BP, blood pressure; eGFR, estimated glomerular filtration rate; FBS, fasting blood glucose; AST, Aspartate aminotransferase; ALT, Alanine aminotransferase; HDL, high-density lipoprotein; LDL, low-density lipoprotein; UACR, urine albumin/creatinine ratio.

**Table 2:** Linear regression of platelet count (10^3^/μL).

Variable	Crude		Model I	
	Slope	*p*	Slope	*p*
Age (years)	−0.2592	<0.0001		
Sex (vs. male)	17.616	<0.0001		
Smoker (vs. non-smoker)	0.8267	0.5235		
Body mass index (kg/m^2^)	1.6425	<0.0001	0.9304	<0.0001
Waist circumference (cm)	0.0704	0.2791		
Systolic BP (mmHg)	−0.0531	0.3193		
Diastolic BP (mmHg)	−0.0857	0.2421		
White blood cell (10^9^/L)	9.6421	<0.0001	8.1295	<0.0001
Hemoglobin (g/dL)	−6.3888	<0.0001	−5.5861	<0.0001
eGFR (mL/min/1.73 m)	0.4311	<0.0001	0.2451	<0.0001
FBS (mg/dL)	0.4811	<0.0001	0.1740	0.0099
Hemoglobin A1c (%)	27.510	<0.0001	17.679	<0.0001
AST (IU/L)	−0.2113	0.0005	−0.0309	0.6284
ALT (IU/L)	−0.0753	0.0189	−0.0052	0.2568
Triglyceride (mg/dL)	0.0543	<0.0001	0.0248	<0.0001
HDL cholesterol (mg/dL)	−0.0635	0.1884		
LDL cholesterol (mg/dL)	0.2079	<0.0001	0.1600	<0.0001
25-vitamin D (ng/mL)	−0.6938	<0.0001	−0.3451	0.0127
UACR (mg/g creatinine)	0.1662	0.0001	0.1436	0.0008

Model I adjusted for age, sex, and smoking history.

**Table 3:** Multivariate logistic regression analysis of microalbuminuria *.

Variable	Crude		Model I		Model II		Model III	
	OR	95% CI	OR	95% CI	OR	95% CI	OR	95% CI
Age (years)	1.034	1.025–1.042						
Sex (vs. male)	1.839	1.404–2.490						
Smoker (vs. non-smoker)	1.333	0.913–1.949						
Body mass index (kg/m^2^)	1.040	0.995–1.087						
Waist circumference (cm)	1.011	0.966–1.026						
Systolic BP (mmHg)	1.026	1.012–1.039	1.021	1.009–1.034				
Diastolic BP (mmHg)	1.013	0.996–1.030						
White blood cell count (10^9^/L)	1.082	0.999–1.170						
Hemoglobin (g/dL)	0.920	0.842–1.006						
Platelet (10^3^/μL)	1.093	1.036–1.154	1.008	1.002–1.014	1.004	1.002–1.006	1.003	1.001–1.005
eGFR (mL/min/1.73 m)	0.994	0.985–1.004						
FBS (mg/dL)	1.039	1.023–1.055	1.030	1.015–1.046	1.026	1.011–1.042		
Hemoglobin A1c (%)	1.543	1.029–2.313	1.001	0.652–1.504				
AST (IU/L)	1.007	1.002–1.012	1.006	1.001–1.011				
ALT(IU/L)	1.001	0.993–1.008						
Triglyceride (mg/dL)	1.001	1.001–1.002	1.001	1.001–1.002				
HDL cholesterol (mg/dL)	0.994	0.983–1.006						
LDL cholesterol (mg/dL)	1.012	1.003–1.020	1.009	1.001–1.017				
25-vitamin D (ng/mL)	1.018	0.992–1.045						

* Defined as UACR (mg/g creatinine) between 30 and 300. Model I, adjusted for age, sex, and smoking history. Model II, adjusted for age, sex, smoking history, systolic BP, AST, triglyceride, and LDL cholesterol. Model III, adjusted for age, sex, smoking history, systolic BP, FBS, AST, triglyceride, and LDL cholesterol. OR, odds ratio; CI, confidence interval.

## Data Availability

The publicly archived datasets can be downloaded through link to the KCDC. https://knhanes.kdca.go.kr/knhanes/eng/index.do (accessed on 1 July 2020).

## Supplementary Materials

The following supporting information can be downloaded at: https://www.mdpi.com/article/10.3390/jpm14010089/s1, Table S1: Reference values for anthropometric variables and laboratory tests.
