# Supplementary material for: Association between Relative Thrombocytosis and Microalbuminuria in Adults with Mild Fasting Hyperglycemia
Source: J Pers Med. 2024 Jan 12;14(1):89. doi: 10.3390/jpm14010089 (PMC10817638; doi:10.3390/jpm14010089)
Supplement: Supplementary file 1 [file jpm-14-00089-s001.zip › jpm-2777564-supplementary.pdf]

Supplemental Table S1. Reference values for anthropometric variables and laboratory tests

Body mass index (kg/m^2)	Pre-obese: 23-24.9, Obese: >25
Waist circumference (cm)	Male: ≥90, Female: ≥85
White blood cell (10^9/L)	3.5-10.0
Hemoglobin (g/dL)	Male: 13.3-17.2, Female: 11.0-15.0
Platelet (10^3/μL)	Male: 131-404, Female: 149-393
FBS (mg/dL)	75-99
Hemoglobin A1c (%)	4.4-6.4
AST (IU/L)	Male: 0-40, Female: 0-32
ALT (IU/L)	Male: 0-41, Female: 0-33
Triglyceride (mg/dL)	40-155
HDL cholesterol (mg/dL)	30-70
LDL cholesterol (mg/dL)	0-142
25-vitamin D (ng/mL)	Deficiency: <20, Insufficiency: 20.0-29.9
UACR (mg/g creatinine)	0-30
